# The mRNA Vaccination Rate Is Negatively and the Proportion of Elderly Individuals Is Positively Associated With the Excess Mortality Rate After 2020 in Japan

**DOI:** 10.7759/cureus.52490

**Published:** 2024-01-18

**Authors:** Dai Ato

**Affiliations:** 1 Research, Independent Researcher, Toyonaka, JPN

**Keywords:** booster shot, partial vaccination rate, proportion of the elderly, sars-cov-2, covid-19

## Abstract

Background: The impact of mRNA vaccines on excess mortality during the COVID-19 pandemic in Japan is not clear. This study aimed to verify the explanatory factors of excess mortality rate using officially published data by government and research institutions.

Methods: Multiple regression analysis was performed using the excess mortality rate in Japanese prefectures as the objective variable and the mRNA vaccination rate, proportion of elderly individuals in the population, number of physicians per population, and medical expenditure per person as explanatory variables.

Results: From July 2021 to April 2023, the independent determinants of the excess mortality rate were as follows: proportion of elderly individuals (regression coefficient (B) = 0.0097, p < 0.001), partial vaccination rate (B = −0.0034, p = 0.048), proportion of elderly individuals (B = 0.010, p < 0.001), and third-shot vaccination rate (B = −0.0025, p < 0.046). The stepwise method did not essentially change the results. However, the p-values were smaller. The other two indicators were not associated with the excess mortality rate.

Conclusions: mRNA vaccination was associated with a lower excess mortality in Japan during the period, whereas the proportion of elderly individuals was associated with an increase in excess mortality. Thus, a policy of aggressive recommendations for mRNA vaccination is justified.

## Introduction

Starting at the beginning of 2020, the COVID-19 pandemic led countries worldwide to implement policies to control its spread. By the end of 2020, an mRNA vaccine was developed and introduced in the United States. In Japan, in early 2021, the vaccination of high-risk individuals and healthcare workers began, followed by the general population. Regardless of national policy efforts, by the end of September 2023, the number of individuals infected with COVID-19 worldwide had risen to approximately 800 million and the number of deaths to approximately seven million [1]. Furthermore, the global mortality rate has increased since 2020 [2].

The number of COVID-19 deaths per population in Japan is lower than in other developed countries [1]. The most likely reason for this was good public adherence to policies, such as vaccination rates and the recommendation to wear sanitary masks. In contrast, Japan showed a slight decline in excess mortality in 2020, but a slight increase after 2021 [3,4].

The main reason for the upward trend in excess mortality is thought to be the reduced ability to cope with other diseases due to the strain on the medical system [5,6]. This may be due to an increase in the number of deaths among particularly vulnerable elderly individuals who are unable to benefit from medical support owing to limited medical resources. This situation varies among prefectures. However, because the strain on the medical system is difficult to assess objectively, a numerical assessment of excess mortality from this effect is difficult. Hence, in the present study, the impact of the medical system strain in each prefecture was replaced by the proportion of elderly individuals in the population. Other indicators examined were the number of physicians per population and medical expenditure per person as medical indicators, along with vaccination rates, which were analyzed as potential explanatory factors for excess mortality.

## Materials and methods

Data and evaluation period

Two separate evaluation periods were considered in this study. The first was the entire period from January 2020 through April 2023. The other was for approximately two years, from July 1, 2021 onward to before the end of April 2023. This is because the excess mortality rate showed a downward trend in 2020 [3], and consideration of the combined impact of the rebound beginning in 2021 was necessary. Furthermore, mRNA vaccination began in early 2021, and an obvious increasing trend in excess mortality was observed beginning in late 2021. If vaccination directly affected the excess mortality rate, this period should be considered separately.

The percentage of elderly individuals in each prefecture was based on published vital statistics at the end of October 2022 [7]. Elderly individuals were defined as those aged 65 years or older, and the proportion of elderly individuals was calculated as the number of elderly individuals divided by the population of each prefecture. Population size was also used to calculate the excess mortality rate.

The number of deaths in each prefecture was obtained from "https://exdeaths-japan.org/" [8]. Data that were available on April 30, 2023 were used. This date was chosen as the deadline because the infection situation had ameliorated, and policy changes to COVID-19 were implemented beginning in May 2023. The number of deaths observed in the prefectures during this period and their estimated values were obtained.

The excess mortality rate (%) was calculated by the following equation:

$100\left ( A - B \right ) / P$.

Where *A* is the total number of observed deaths in each prefecture in the period, *B* is the total number of estimated deaths in each prefecture in the period, and *P* is the population of each prefecture.

The vaccination rate for each prefecture was obtained from a website provided by Sapporo Medical University School of Medicine [9]. Figures that were available on April 30, 2023 were used. The reason for setting a deadline for this date was the same as described above. The vaccination rate primarily used was the partial vaccination rate, which means that vaccination was performed at least once. For booster vaccinations, the third-shot vaccination rate was used as a representative value. In addition, the fourth-shot vaccination rate was used in multiple regression analysis for confirmation (see the Statistical analysis section for the explanation).

The number of physicians per population (100,000) was used as the medical indicator. The figures were obtained from the Ministry of Health, Labour and Welfare's website and used for fiscal year 2020 [10]. The medical expenditure per person (the ratio to the national average) was also used as a surrogate indicator for the medical index and economic strength of each prefecture. These data were also obtained from the Ministry of Health, Labor, and Welfare [11]. Note that this indicator was used for fiscal year 2019 to account for possible changes before and after the COVID-19 pandemic.

Statistical analysis

EZR version 1.61 was used for analysis [12]. Means and standard deviations were calculated for each variable. Correlations for each variable were calculated using Pearson's correlation coefficient test. All correlation coefficients between the excess mortality rates and other variables were calculated. Correlations between vaccination rates were also calculated. After confirming the correlation values, a multiple linear regression analysis was performed with the excess mortality rate as the objective variable. Candidate explanatory variables, such as vaccination rate, proportion of elderly individuals in the population, number of physicians per population, and medical expenditure per person were included in the model for the multiple linear regression analysis. In the multiple regression model, an additional stepwise Akaike information criterion (AIC) was performed.

In multiple regression analysis, the study period and vaccination rates were divided into two patterns as follows: period 1, from January 2020 through April 2023 (entire period); period 2, from July 2021 through April 2023 (after July 2021); vaccination rate 1: partial vaccination rate; vaccination rate 2: third-shot vaccination rate.

The reason for analyzing vaccination rates separately is that they cancel each other out when they are simultaneously entered into the multiple regression analysis. This is due to the high correlation between the two (as shown in the Results section). Furthermore, as discussed in more detail in the Results section, the fourth-shot vaccination rate showed a weak (non-significant) positive correlation with excess mortality after July 2021, which was also analyzed separately. P-values of ≤ 0.05 were considered statistically significant (two-tailed).

Ethics statement

There are no ethical restrictions in Japan when using statistical data that cannot identify personal information. This study uses publicly available statistics only. Therefore, ethics approval is exempted. Nevertheless, the author is committed to this research with ethics.

## Results

Table 1 shows the variables for the 47 prefectures in Japan. The excess mortality rates were 0.14 ± 0.033% (for the entire period) and 0.16% ± 0.039% (after July 2021). The proportion of elderly individuals was 31.49 ± 3.06%, the partial vaccination rate was 79.30 ± 3.47%, and the third-shot vaccination rate was 70.88 ± 4.92%. The number of physicians per population and medical expenditure per person were 199.7 ± 35.0 and 1.05 ± 0.10, respectively.

**Table 1:** Variables for 47 prefectures in Japan Medical expenditure per person is used as the ratio to the national average. The entire period: January 2020 through April 2023. After July 2021: July 2021 through April 2023.

Variables	Mean ± standard deviation
Excess mortality rate (entire period) (％)	0.14 ± 0.033
Excess mortality rate (after July 2021) (％)	0.16 ± 0.039
Partial vaccination rate (％)	79.30 ± 3.47
Third-shot vaccination rate (％)	70.88 ± 4.92
Fourth-shot vaccination rate (％)	49.39 ± 5.92
Proportion of elderly individuals (％)	31.49 ± 3.06
Number of physicians (per 100,000)	199.7 ± 35.0
Medical expenditure per person	1.05 ± 0.10

The correlation coefficients of each variable with excess mortality rates are shown in Table 2. Throughout the study period, the partial vaccination rate showed a significant negative correlation. The proportion of elderly individuals, number of physicians per population, and medical expenditure per person were significantly positively correlated.

**Table 2:** Pearson correlation coefficients between the excess mortality rate and each variable Entire period: January 2020 through April 2023. After July 2021: July 2021 through April 2023. Medical expenditure per person is used as the ratio to the national average.

Entire period or after July 2021	Excess mortality rate (after July 2021) (%)	Partial vaccination rate (%)	Third-shot vaccination rate (％)	Fourth-shot vaccination rate (％)	Proportion of elderly individuals (％)	Number of physicians (per 100,000)	Medical expenditure per person
Excess mortality rate (entire period) (%)	r = 0.77, p < 0.001	r =0.33, p = 0.026	r = -0.23, p = 0.12	r = -0.17, p = 0.25	r = 0.38, p < 0.009	r = 0.49, p < 0.001	r = 0.39, p < 0.007
Excess mortality rate (after July 2021) (%)	ー	r = 0.048, p = 0.76	r = 0.079, p = 0.60	r = 0.17, p = 0.24	r = 0.62, p < 0.001	r = 0.43, p < 0.003	r = 0.44, p < 0.002

Neither partial nor third-shot vaccination rates showed a significant correlation with the excess mortality rate after July 2021. However, the third-shot vaccination rate was insignificantly positively correlated with the excess mortality rate (r = 0.079; p = 0.60). The fourth-shot vaccination rate was also insignificantly positively correlated with excess mortality (r = 0.17; p = 0.24). Furthermore, the proportion of elderly individuals moderately correlated with the excess mortality rate (r = 0.62; p < 0.001).

The number of physicians per population and the medical expenditure per person in each period showed moderate correlations with the excess mortality rate (ｒ= 0.39 - 0.49; all p＜ 0.01).

The correlations for each vaccination rate are shown in Table 3. Each of these showed a high correlation.

**Table 3:** Pearson correlation coefficients between each vaccination rate

Partial or third-shot vaccination rate (％)	Third-shot vaccination rate (％)	Fourth-shot vaccination rate (％)
Partial vaccination rate (％)	r = 0.94, p < 0.001	r = 0.88, p < 0.001
Third-shot vaccination rate (％)	ー	r = 0.97, p < 0.001

The third and fourth-shot rates and the proportion of the elderly were moderately significantly correlated (r = 0.57 and 0.68, respectively; both p < 0.001). The number of physicians per population and medical expenditures per person showed a moderate correlation (r = 0.55, p < 0.001).

The results of the multiple linear regression analysis for excess mortality for the entire period are shown in Table 4. The vaccination rates were negative determinants, and the proportion of elderly individuals was a positive determinant (regression coefficient (B) = −0.0047 and p < 0.004 for the partial vaccination rate; B = 0.0062 and p < 0.003 for the proportion of elderly individuals; B = −0.0037 and p ＜ 0.002 for the third-shot vaccination rate; and B ＝ 0.0068 and ｐ＜ 0.002 for the proportion of elderly individuals).

**Table 4:** Multiple linear regression analysis for the excess mortality rate as a dependent variable (entire period) Entire period: January 2020 through April 2023. AIC: Akaike information criterion. In the model of partial vaccination rate selected: Multiple linear regression model: R-squared = 0.4337, p < 0.001; stepwise method: R-squared = 0.4175, p < 0.001. In the model of three-time vaccination rate selected: Multiple linear regression model: R-squared = 0.4566, p < 0.001; stepwise method: R-squared = 0.4359, p < 0.001.

Selected vaccination rate	Variables	Multiple linear regression			Stepwise method (AIC)		
		Coefficient	t value	p	Coefficient	t value	p
Partial vaccination rate	Partial vaccination rate	-0.0047	-3.12	<0.004	-0.0054	-4.55	<0.001
	Proportion of elderly individuals	0.0062	3.24	<0.003	0.0065	4.85	<0.001
	Number of physicians	0.00016	1.09	0.28	-	-	-
	Medical expenditure per person	-0.026	-0.48	0.63	-	-	-
Third-shot vaccination rate	Third-shot vaccination rate	-0.0037	-3.45	<0.002	-0.0044	-4.78	<0.001
	Proportion of elderly individuals	0.0068	3.53	<0.002	0.008	5.46	<0.001
	Number of physicians	0.00016	1.12	0.27	-	-	-
	Medical expenditure per person	0.0021	0.039	0.97	-	-	-

Table 5 presents the results of the multiple linear regression analysis for the excess mortality rate after July 2021. The results were similar to those for the entire period (B = −0.0034 and p = 0.048 for the partial vaccination rate; B = 0.0097 and p < 0.001 for the proportion of elderly individuals; B = −0.0025 and ｐ= 0.046 for the third-shot vaccination rate; and B＝0.010 and ｐ＜ 0.001 for the proportion of elderly individuals).

**Table 5:** Multiple linear regression analysis for the excess mortality rate as a dependent variable (after July 2021) After July 2021: July 2021 through April 2023. AIC: Akaike information criterion. In the model of partial vaccination rate selected: Multiple linear regression model: R-squared = 0.4997, p < 0.001; stepwise method: R-squared = 0.484, p < 0.001. In the model of third-shot vaccination rate selected: Multiple linear regression model: R-squared = 0.5008, p < 0.001; stepwise method: R-squared = 0.4856, p < 0.001. In the model of fourth-shot vaccination rate selected: Multiple linear regression model: R-squared = 0.5036, p < 0.001; stepwise method: R-squared = 0.4881, p < 0.001.

Selected vaccination rate	Variables	Multiple linear regression			Stepwise method (AIC)		
		Coefficient	t value	p	Coefficient	t value	p
Partial vaccination rate	Partial vaccination rate	-0.0034	-2.04	0.048	-0.0039	-3	<0.005
	Proportion of elderly individuals	0.0097	4.57	<0.001	0.0096	6.41	<0.001
	Number of physicians	0.00017	1.05	0.3	-	-	-
	Medical expenditure per person	-0.048	-0.79	0.44	-	-	-
Third-shot vaccination rate	Third-shot vaccination rate	-0.0025	-2.06	0.046	-0.0031	-3.02	<0.005
	Proportion of elderly individuals	0.01	4.55	<0.001	0.011	6.4	<0.001
	Number of physicians	0.00018	1.13	0.26	-	-	-
	Medical expenditure per person	-0.027	-0.45	0.65	-	-	-
Fourth-shot vaccination rate	Fourth-shot vaccination rate	-0.0024	-2.12	0.04	-0.0029	-3.07	<0.004
	Proportion of elderly individuals	0.011	4.46	<0.001	0.012	6.27	<0.001
	Number of physicians	0.00018	1.15	0.26	-	-	-
	Medical expenditure per person	-0.027	-0.46	0.65	-	-	-

Table 5 presents the results obtained when the fourth-shot vaccination rate was used. It was similar to the results described above (B = −0.0024 and p = 0.040 for the fourth-shot vaccination rate and B ＝ 0.011 and ｐ＜ 0.001 for the proportion of elderly individuals).

Furthermore, Tables 4, 5 show that the stepwise method using AIC did not change the results. However, the p-values were smaller.

In all multiple linear regression analyses, neither the number of physicians per population nor medical expenditure per person were significant explanatory variables. Furthermore, the results of the multiple regression analysis were essentially unchanged when the number of physicians per population and medical expenditures per person were either one or had no inputs (data not shown).

## Discussion

To the best of our knowledge, this is the first study to show that the mRNA vaccination rate was an independent negative determinant and that the proportion of elderly individuals was an independent positive determinant of the excess mortality rate since the start of the COVID-19 pandemic in Japan. The analysis consistently showed a strong association between the excess mortality rate and the proportion of elderly individuals, followed by the mRNA vaccination rate.

In the data after July 2021, the Pearson correlation analysis showed a non-significant positive correlation between the third- and fourth-shot vaccination rates and the excess mortality rate. It is considered that the higher risk of the elderly population compared to young and middle-aged individuals underlies this relationship and the resulting increase in vaccination rates.

The number of physicians per population and medical expenditure were not explanatory factors for excess mortality. This may be because social constraints and medical system strain caused the collapse of the healthcare system in Japan, resulting in excess mortality.

According to the current results, a 1% increase in the partial vaccination rate would have resulted in a decrease in excess mortality of approximately 0.005%, a reduction of five per 100,000 people over the entire period, or a reduction of three per 100,000 people in about two years after July 2021.

There were no clear differences in the impact of vaccination and the proportion of elderly individuals on excess mortality over the entire period. However, after July 2021, the impact of the vaccination rate weakened slightly compared to that of the proportion of elderly individuals, whereas the impact of the proportion of elderly individuals became more pronounced. This was clear in the stepwise method using AIC. This suggests that during periods of higher excess mortality, the burden resulting from the medical system strain was manifested more strongly in the elderly population, even beyond the effect of vaccination, resulting in excess deaths caused by various diseases [4,5].

The present results are consistent with those of previous studies [13-15]. The first report of mRNA vaccine efficacy was published in Israel [16]. Since then, there have been variations in the viral epidemic strains, but a certain level of efficacy has been maintained [14,15,17,18]. Furthermore, several studies, including the results of a meta-analysis, have shown that vaccination reduces not only COVID-19-related but also all-cause mortality [13-15].

Mendoza-Cano et al. showed a negative correlation between the excess mortality rate and worldwide vaccination rate [13]. This study demonstrated similar results using the data of each prefecture in Japan, the negative association between the vaccination rate, and the excess mortality.

This study is based on highly objective data compiled by the Japanese government and research institutions and further supports existing vaccine efficacy research findings.

Various types of disinformation regarding COVID-19 and the efficacy of mRNA vaccines have spread worldwide. The JAMA Network published an analysis of the issue of misinformation-transmitting physicians [19]. In the US, many messages were sent, especially via the X social media platform (previously “Twitter”). This is also the case in Japan. Frequent hoaxes were observed, especially on Twitter, and the resulting health risks to the public and the discrediting of the government and medical personnel were serious.

This study demonstrated that mRNA vaccination reduces excess mortality. Hopefully, the present findings will help provide objective explanations for the effectiveness of the vaccine and its appeal to the public.

This study had strengths and limitations. In this study, highly objective numerical values were used, and multiple patterns of multiple linear regression analyses were performed to confirm the results. Therefore, a certain degree of reliability was achieved in terms of statistical accuracy. One limitation is that this study was only a verification of the association and not a proof of causality. Furthermore, the highest explanatory power of multiple regression analysis for the excess mortality rate is approximately 50%, and it is considered that medical system strain accounts for most of the approximately 50% of the undetermined factors. However, it is reasonable to assume that mRNA vaccination contributes not only to infection prevention but also to a subsequent reduction in excess mortality and that the proportion of elderly individuals is a major factor in the increase in excess mortality.

## Conclusions

During the post-2020 COVID-19 pandemic period in Japan, the mRNA vaccination rate was an independent determinant of controlling excess mortality. The proportion of elderly individuals in the population is an independent determinant of increased excess mortality.

## References

[1] (2023). WHO COVID-19 dashboard. https://covid19.who.int/.

[2] (2023). WHO. The true death toll of COVID-19: estimating global excess mortality. https://www.who.int/data/stories/the-true-death-toll-of-covid-19-estimating-global-excess-mortality.

[3] Onozuka D, Tanoue Y, Nomura S (2022). Reduced mortality during the COVID-19 outbreak in Japan, 2020: a two-stage interrupted time-series design. Int J Epidemiol.

[4] Nomura S, Eguchi A, Tanoue Y, Yoneoka D, Kawashima T, Suzuki M, Hashizume M (2022). Excess deaths from COVID-19 in Japan and 47 prefectures from January through June 2021. Public Health.

[5] Nomura S, Eguchi A, Ghaznavi C (2023). Changes in cerebrovascular disease-related deaths and their location during the COVID-19 pandemic in Japan. Public Health.

[6] Uchi Y, Yamashita E, Kami M, Takita M (2023). Changes in the cause of death in Japan before and during the COVID-19 pandemic. Arch Gerontol Geriatr.

[7] (2023). Ministry of Internal Affairs and Communications. Estimate of the population. https://www.stat.go.jp/data/jinsui/2022np/index.html.

[8] (2023). Dashboard of excess and exiguous deaths in Japan. https://exdeaths-japan.org/graph/weekly/.

[9] (2023). Trends in new coronavirus vaccination rate in each prefectures. https://web.sapmed.ac.jp/canmol/coronavirus/japan_vaccine.html?a=1&d=0.

[10] (2023). Ministry of Health, Labour and Welfare. Research of medical facility. https://www.mhlw.go.jp/toukei/saikin/hw/iryosd/20/.

[11] (2023). Ministry of Health, Labour and Welfare. Analysis of regional differences in medical expenses. https://www.mhlw.go.jp/stf/seisakunitsuite/bunya/kenkou_iryou/iryouhoken/database/iryomap/index.html.

[12] Kanda Y (2013). Investigation of the freely available easy-to-use software 'EZR' for medical statistics. Bone Marrow Transplant.

[13] Mendoza-Cano O, Trujillo X, Huerta M (2023). Assessing the influence of COVID-19 vaccination coverage on excess mortality across 178 countries: a cross-sectional study. Vaccines (Basel).

[14] Hoxha I, Agahi R, Bimbashi A (2022). Higher COVID-19 vaccination rates are associated with lower COVID-19 mortality: a global analysis. Vaccines (Basel).

[15] Yang XH, Bao WJ, Zhang H, Fu SK, Jin HM (2023). The efficacy of SARS-CoV-2 vaccination in the elderly: a systemic review and meta-analysis. [PREPRINT]. J Gen Intern Med.

[16] Dagan N, Barda N, Kepten E (2021). BNT162b2 mRNA COVID-19 vaccine in a nationwide mass vaccination setting. N Engl J Med.

[17] Abu-Raddad LJ, Chemaitelly H, Ayoub HH (2022). Effect of mRNA vaccine boosters against SARS-CoV-2 Omicron infection in Qatar. N Engl J Med.

[18] Maeda H, Saito N, Igarashi A (2023). Effectiveness of mRNA COVID-19 vaccines against symptomatic SARS-CoV-2 infections during the SARS-CoV-2 Omicron BA.1 and BA.2 epidemic in Japan: vaccine effectiveness real-time surveillance for SARS-CoV-2 (VERSUS). Expert Rev Vaccines.

[19] Sule S, DaCosta MC, DeCou E, Gilson C, Wallace K, Goff SL (2023). Communication of COVID-19 misinformation on social media by physicians in the US. JAMA Netw Open.

